# Association of *JAK2*V617F allele burden and clinical correlates in polycythemia vera: a systematic review and meta-analysis

**DOI:** 10.1007/s00277-024-05754-4

**Published:** 2024-04-23

**Authors:** Chih-Cheng Chen, Justin L. Chen, Alex Jia-Hong Lin, Lennex Hsueh-Lin Yu, Hsin-An Hou

**Affiliations:** 1 Division of Hematology and Oncology, Department of Medicine, Chang Gung Memorial Hospital, Chiayi, 613 Taiwan; 2 College of Medicine, Chang Gung University, Taoyuan, 333 Taiwan; 3 Medical Affairs Department, Panco Healthcare Co., Ltd., A Pharmaessentia Company, Taipei, 115 Taiwan; 4 Division of Hematology, Department of Internal Medicine, National Taiwan University Hospital, Taipei, 100 Taiwan; 5 Division of General Medicine, Department of Internal Medicine, National Taiwan University Hospital, Taipei, 100 Taiwan

**Keywords:** Polycythemia vera, Janus kinase 2 V617F, Allele burden, Variant allele frequency

## Abstract

Janus kinase 2 (*JAK2*) V617F mutation is present in most patients with polycythemia vera (PV). One persistently puzzling aspect unresolved is the association between *JAK2*V617F allele burden (also known as variant allele frequency) and the relevant clinical characteristics. Numerous studies have reported associations between allele burden and both hematologic and clinical features. While there are strong indications linking high allele burden in PV patients with symptoms and clinical characteristics, not all associations are definitive, and disparate and contradictory findings have been reported. Hence, this study aimed to synthesize existing data from the literature to better understand the association between *JAK2*V617F allele burden and relevant clinical correlates. Out of the 1,851 studies identified, 39 studies provided evidence related to the association between *JAK2*V617F allele burden and clinical correlates, and 21 studies were included in meta-analyses. Meta-analyses of correlation demonstrated that leucocyte and erythrocyte counts were significantly and positively correlated with *JAK2*V617F allele burden, whereas platelet count was not. Meta-analyses of standardized mean difference demonstrated that leucocyte and hematocrit were significantly higher in patients with higher *JAK2*V617F allele burden, whereas platelet count was significantly lower. Meta-analyses of odds ratio demonstrated that patients who had higher *JAK2*V617F allele burden had a significantly greater odds ratio for developing pruritus, splenomegaly, thrombosis, myelofibrosis, and acute myeloid leukemia. Our study integrates data from approximately 5,462 patients, contributing insights into the association between *JAK2*V617F allele burden and various hematological parameters, symptomatic manifestations, and complications. However, varied methods of data presentation and statistical analyses prevented the execution of high-quality meta-analyses.

**Supplementary Information:**

The online version contains supplementary material available at 10.1007/s00277-024-05754-4.

## Introduction

Polycythemia vera (PV), along with essential thrombocythemia and primary myelofibrosis, constitute the classic Philadelphia-negative myeloproliferative neoplasms (MPNs), a group of rare hematologic cancers characterized by the overproduction of one or more blood cell types. PV is typically marked by erythrocytosis, and in many cases concurrent leukocytosis and thrombocytosis. The excessive levels of blood cells result in blood thickening and a reduction in blood flow, elevating the risk of symptoms such as hemorrhage and thrombosis. These complications significantly impact the quality of life and in severe cases can be fatal. As early-stage patients are often asymptomatic for many years, and the symptoms of PV lack distinct features, suspicion of PV and subsequent diagnosis frequently occur later, following the exclusion of other diseases.

In 2005, several research groups independently identified a mutation in the Janus kinase 2 (*JAK2*) gene, *JAK2*V617F, that revolutionized the diagnosis and treatment of MPNs [1–4]. JAK2, a member of the Janus family of nonreceptor tyrosine kinases, plays a crucial role in hematopoiesis. Upon binding to associated receptor molecules, JAK2 induces conformational changes that phosphorylate specific tyrosine residues on the intracellular domain of the receptor, creating docking sites for specific signaling molecules [5]. The *JAK2*V617F mutation removes the intrinsic inhibitory mechanism and results in the overactivation of the *JAK2* protein. This leads to constitutional activation of its receptors, aberrant downstream signaling, and an increase in hematopoiesis [6]. This mutation is present in 95% of patients with PV and 50–60% of patients with essential thrombocythemia or primary myelofibrosis [7]. The remaining PV patients without the mutation often harbor other mutations located on exon 12 of *JAK2* [8]. Therefore, the involvement of *JAK2* mutations in PV underpins its significance in this disease.

One persistently puzzling aspect in PV that remains somewhat unresolved is the association between *JAK2*V617F allele burden (or variant allele frequency) and the relevant clinical characteristics. Numerous studies have reported associations between allele burden and both hematologic and clinical features of MPNs. For instance, a high allele burden has been correlated with increases in thrombosis and disease transformation [9]. While there are strong indications linking high allele burden in PV patients with symptoms and clinical characteristics, not all associations are definitive, and disparate and contradictory findings have been reported. To the best of our knowledge, a meta-analysis has yet to be conducted to investigate the association between *JAK2*V617F allele burden and the clinical characteristics of PV. Hence, this study aimed to synthesize existing data from the literature to better understand the association between *JAK2*V617F allele burden and relevant clinical correlates.

## Methods

### Eligibility criteria, information sources and search strategy

This systematic review and meta-analysis study adhered to the Preferred Reporting Items for Systematic Review and Meta-analyses (PRISMA) guidelines [10]. This review included studies published between January 1st, 2005, and February 28th, 2022, where patients were diagnosed with PV, *JAK2*V617F allele burden was quantified, and hematologic parameters and/or clinical outcomes were measured. Only original research articles were considered.

This systematic review was conducted across three databases, namely PubMed, Science Direct, and Wiley Online Library. The following search terms were utilized: (“JAK2” OR “JAK2V617F” OR “V617F”) AND (“allele burden” OR “allelic burden” OR “clonal dominance” OR “variant allele frequency”) AND (“Polycythemia” OR “Polycythaemia”). Exclusion criteria encompassed studies not published in English, those lacking full-text, duplicate studies, and reviews.

### Selection process and data collection process

Two authors (JLC & AJL) independently screened the title and abstract of each study for initial inclusion in our systematic review. Studies upon which both authors reached consensus were included. Any disagreements were resolved through discussion or by a third author if necessary. Two authors independently reviewed the full text (JLC & LHY) for data applicability.

Only studies presenting data pertaining to a hematologic parameter or clinical outcome correlating with a quantified *JAK2*V617F allele burden were included. Discrepancies were resolved through discussion or by a third author if necessary. Both title and abstract screening as well as full-text review were conducted on the Covidence platform (app.covidence.org). Data extraction was performed using a standardized form in Microsoft Excel by one author (JLC), with the accuracy of the extracted data verified by two authors (CCC & HHA).

### Data items

Peripheral blood or bone marrow samples from patients were taken at the time of diagnosis or during follow-up. Some patients were on treatments for PV, which included aspirin, phlebotomy, and/or cytoreductive agents. *JAK2* allele burden was measured using validated methods. Various clinical outcomes and hematologic parameters were assessed. Continuous variables extracted included red blood cell count (RBC), platelet count (PLT), white blood cell count (WBC), hematocrit (Hct), hemoglobin (Hb), spleen size, and *JAK2*V617F allele burden. Count variables extracted included splenomegaly, pruritus, thrombosis, hemorrhage, post-PV transformation to myelofibrosis (MF), and post-PV transformation to acute myeloid leukemia (AML). The mean and standard deviation of the continuous variables were extracted. Correlation coefficients and sample sizes were extracted where Pearson or Spearman correlation tests were performed. The following information was also extracted: surname of first author, year of publication, country of study site, sample size, source of DNA, *JAK2*V617F quantification method, sample collection time point, *JAK2*V617F allele burden data presentation, and applied statistical methods.

### Study risk of bias assessment

Two authors (JLC & LHY) independently evaluated the quality of studies using critical appraisal checklists from the Joanna Briggs Institute (JBI) [11]. The criteria included the following items: (1) Were the criteria for inclusion in the sample clearly defined?; (2) Were the study subjects and the setting described in detail?; (3) Was the exposure measured in a valid and reliable way?; (4) Were objective, standard criteria used for measurement of the condition?; (5) Were confounding factors identified?; (6) Were strategies to deal with confounding factors stated?; (7) Were the outcomes measured in a valid and reliable way?; and (8) Was appropriate statistical analysis used?. Each item received a response of “Yes,” “No,” or “Unclear,” corresponding to 1, 0, or 0 points, respectively. Studies consistent between the two authors with fewer than three items marked as “No” or “Unclear” were included in the systematic review and meta-analysis. Disagreements between authors were resolved through discussion or by involving a third author if needed.

### Effect measures and synthesis methods

Qualitative descriptions and summaries of evidence were provided, and meta-analyses were conducted using Comprehensive Meta-Analysis 3.0. Pooled odds ratio (OR), standardized mean difference (SMD), correlation coefficients, 95% confidence intervals (95%CI), and standard error (SE) were calculated using the software.

Due to the diversity of the included data, we categorized them based on how *JAK2*V617F allele burden was presented: (a) *JAK2*V617F allele burden tested against another variable using correlation tests; (b) patients grouped by *JAK2*V617F allele burden level, with mean values and standard deviations of their clinical characteristics presented; or (c) patients grouped by *JAK2*V617F allele burden level, with count data presented for their clinical measurements (e.g. record of later MF transformation). For uniformity, continuous variables were converted into the same units (e.g. 10^9/ml).

All included studies following full-text review were tabulated (Table 1). Meta-analyses were depicted as forest plots (Figs. 2, 3 and 4). Random effects models were employed to address heterogeneity in all meta-analyses.

**Table 1:** Study details on JAK2V617F allele burden measurement and data presentation with clinical correlates

Author [ref]	Year	Country	N	DNA source	JAK2V617F quantification	Samples at dx	Data presentation	Clinical correlates	Statistical methods
Alvarez- Larran	2014	Spain	163	Granulocytes	AS-qPCR	Some	Dichotomous (50%)	Thrombosis; MF transformation	KM analysis; multivariate poisson regression
Bai	2015	China	272	PB granulocytes	AS-qPCR	All	Dichotomous (50%)	PLT; WBC; Thrombosis; MF transformation	Independent t-test; MWU test; KM analysis; univariate/multivariate Cox regression
Barbui	2011	Italy	71	PB	Not stated	Some	Dichotomous (50%)	High-sensitivity C-reactive protein; pentraxin-3	Multivariate regressions; univariate/multivariate logistic regressions
Bellanne- Chantelot	2006	Belgium, France	81	PB mononuclear cells; BM cells; EEC	PCR	No	Dichotomous (50%)	Disease duration; Hb; Hct; PLT; WBC; hematological complications; splenomegaly; thrombosis; OS; EEC status	Chi-square test; Fisher exact test; KW test; KM analysis
Carobbio	2009	Italy	415	Not stated	qRT-PCR	Some	Trichotomous (1–25%;26–50%;>50%)	Thrombosis	Chi-square test; Fisher exact test; WRS test; KM analysis; test for trend of the survivor function; multivariate Cox regression
Cokic	2015	Serbia	92	PB granulocytes	PCR	All	Dichotomous (50%)	Hb; MCV; RBC; PLT; WBC; CD34 + cell count	Independent t-test
Coucelo	2014	Portugal	31	PB	AS-qPCR; qRT-PCR	Not stated	Continuous	PMN aggregates; CD11b expressions; CD63 expression; monocyte TF expression	Spearman correlation; t-test
Edahiro	2014	Japan	66	PB mononuclear cells	ABC-PCR; AS-qPCR	All	Continuous	Hb; PLT; WBC	Pearson correlation
Ferdowsi	2015	Iran	20	PB leukocytes	qRT-PCR	All	Continuous	Hb; PLT; WBC	Spearman correlation
Ferdowsi	2016	Iran	51	PB leukocytes	qRT-PCR	Some	Continuous	Hb; PLT; WBC	Spearman correlation
Gangat	2008a	USA	418	BM	qRT-PCR	All	Dichotomous (pruritis yes/no)	Pruritus	Raw data
Gangat	2008b	USA	137	BM	AS-qPCR	All	Trichotomous (1–25%;26–50%; 51–75%)	Cytogenetics	Undetermined
Gangemi	2012	Italy	20	PB	AS-qPCR	Not stated	Continuous	WBC; pruritus	Spearman correlation
Guglielmelli	2021	Italy	865	Not stated	Not stated	All	Dichotomous (50%)	Age; gender; history of thrombosis; history of hemorrhage; Hb; Hct; PLT; WBC; LDH; pruritus; splenomegaly; thrombosis; MF transformation; leukemic transformation	Chi-square test; MWU test; KM analysis; Cox proportional hazard regression (univariate and multivariate)
Ha	2012	South Korea	22	BM	Pyrosequencing	All	Quaternary (1–25%; 25–50%, 50–75%, 75–100%)	Age; Hb; Hct; PLT; WBC; neutrophil count; organomegaly; thrombotic event; fibrosis	Independent t-test; one-way ANOVA
Hu	2017	China	20	BM; PB	AS-qPCR	All	Continuous	PLT	Pearson correlation
Koren- Michowitz	2012	Israel	101	PB mononuclear cells	Quantitative chip-based MALDI-TOF MS	Not stated	Continuous; dichotomous (clinical correlates)	Gender; ethnicity; disease duration; spleen size; sympotoms; pruritus; erythromelalgia; vascular complications; disease transformations; cytoreductive therapy	Spearman correlation; WRS test
Larsen	2007	Denmark	95	PB leukocytes	qRT-PCR	Some	Continuous; dichotomous (50%)	Age; gender; disease duration; Hb; Hct; PLT; WBC; CD34; LDH; thrombosis; cytoreductive therapy; PRV-1	Spearman correlation; WRS test
Lee	2021	South Korea	61	PB	Direct sequencing	Not stated	Dichotomous (58%)	Age at diagnosis; gender; Hb; Hct; PLT; WBC; LDH; thrombosis at diagnosis and follow-up; MF progression; OS	Chi-square test; MWU test; KM analysis; univariate/multivariate logistic regressions
Lekovic	2017	Serbia	30	PB granulocytes	AS-qPCR; fragment analysis	Some	Dichotomous (50%)	Plasma vascular endothelial growth factor; plasma basic fibroblast growth factor; plasma interleukin-8	Independent t-test
Malak	2012	Belgium, France	97	PB mononuclear cells	Direct sequencing; PCR	Some	Dichotomous (50%)	MF transformation; leukemic transformation	Chi-square test; Fisher exact test
Maslah	2022	France	129	PB whole blood	AS-qPCR	Not stated	Continuous	RCM	Correlation test
Moliterno	2008	USA	138	PB neutrophils, CD4 + cells	AS-qPCR	Some	Continuous; dichotomous (clonal dominance)	Disease duration; PLT; WBC; reticulocyte; spleen size	Correlation test; Independent t-test
Okabe	2016	Japan	74	BM or PB mononuclear cells	MB-PCR; direct sequencing	Not stated	Continuous; dichotomous (70%)	Age; gender; history of thrombosis; history of hemorrhage; vascular risk factors; Hb; Hct; RBC; PLT; WBC; leukocyte ALP; Vitamin 12; chromosomal abnormality; MF transformation; leukemic transformation; therapy	Chi-square test, Fisher’s exact test, and independent t-test; Pearson correlation
Passamonti	2010	Italy	338	Granulocytes	qRT-PCR	Some	Continuous; dichotomous (50%)	Disease duration; Hb; PLT; WBC; pruritus; spleen size thrombosis; hemorrhage; MF transformation; leukemic transformation; MF-free survival; (age-adjusted) BM cellularity	Spearman correlation; KM test; Multivariate Cox regression
Payzin	2014	Turkey	81	Not stated	AS-qPCR	Some	Continuous; dichotomous (50%)	Age; Hb; PLT; WBC; LDH; spleen size	Undetermined
Pieri	2009	Italy	78	PB neutrophils, basophils	ARMS-PCR; QRT-PCR	Not stated	Continuous; dichotomous (50%)	Basophil count; CD63 + basophil count; pruritus; history of thrombosis	Spearman correlation; MWU test
Popova- Labachevska	2019	Macedonia	17	Not stated	AS-qPCR	Not stated	Trichotomous (< 10%;10–50%; >50%)	Age; Hb; Hct; RBC; PLT; WBC	KW test
Ruella	2013	Italy	78	PB leukocytes	AS-qPCR	Some	Dichotomous (50%)	Telomere length	Multivariate ANCOVA
Sacco	2020	Italy	48	PB mononuclear cells	qRT-PCR	Not stated	Continuous	Age; gender; RBC; PLT; VWF: Act; VWF: Ag; HU treatment; splenomegaly	Spearman correlation
Sazawal	2019	India	90	PB granulocytes	qRT-PCR	Not stated	Dichotomous (clinical correlates)	Age; gender; Hb; Hct; PLT; WBC; splenomegaly; thrombosis	Undetermined
Silver	2011	USA	105	PB leukocytes	ARMS-PCR; pyrosequencing	Some	Dichotomous (MF grade; thrombosis type); quarternary (spleen size); quinary (0–20%; 21–40%; 41–60%; 61–80%; 81–100%)	Disease duration; WBC; spleen size; thrombosis type; MF grade	Independent t-test; ANOVA
Stein	2011	USA	161	PB	AS-qPCR	Some	Dichotomous (50%); OR	Gender; thrombosis	Multivariate logistic regression
Stein	2013	USA	204	PB	AS-qPCR	Not stated	Dichotomous (age)	Age	WRS test
Tefferi	2007	USA	186	BM	qRT-PCR	Some	Continuous	Age; gender; Hb; PLT; WBC; pruritus; microvascular symptoms; palpable splenomegaly; major thrombosis; thrombosis type	Multivariate linear regression
Vannucchi	2007a	Italy	173	PB granulocytes	ARMS-PCR; QRT-PCR	All	Continuous; quaternary (1–25%; 25–50%, 50–75%, 75–100%); RR	Gender; Hct; MCV; PLT; WBC; neutrophil count; LDH; leukocyte ALP; erythropoietin; ferritin; systemic symptoms; pruritus; splenomegaly; spleen size; thrombosis; therapy; cytoreduction-free survival; PRV-1	Chi-square test; Fisher exact test; MWU test; KW test; Spearman correlation; KM test; logistic regression
Vannucchi	2007b	Italy	323	PB granulocytes	AS-qPCR	Some	Continuous; dichotomous (50%)	Age; gender; disease duration; Hct; PLT; WBC; systemic symptoms; pruritus; splenomegaly; CV event; therapy	Chi-square test, Fisher’s exact test, and independent t-test; Cox regressions; logistic regressions
Zhao	2016	China	54	BM mononuclear cells	AS-qPCR	All	Continuous; dichotomous (clinical correlates)	Age; gender; Hb; PLT; WBC; splenomegaly; thrombosis	Chi-square test; Pearson correlation; Independent t-test; MWU test; KM test; Cox regression
Zhou	2013	China	57	BM; PB cells	AS-qPCR	Not stated	Continuous	RBC; PLT; WBC; erythropoiesis; granulopoiesis	Spearman correlation

ABC-PCR: alternately binding probe competitive polymerase chain reaction; ANCOVA: analysis of covariance; ANOVA: analysis of variance; ALP: alkaline phosphatase; ARMS-PCR: amplification-refractory mutation sequencing polymerase chain reaction; AS-qPCR: allele specific real-time polymerase chain reaction; BM: bone marrow; dx: diagnosis; CD: cluster of differentiation; CV: cardiovascular; EEC: endogenous erythroid colony; Hb: hemoglobin; Hct: hematocrit; KM: Kaplan-Meier; KW: Kruskal-Wallis; LDH: lactate dehydrogenase; MALDI-TOF MS: matrix-assisted laser desorption-time-of-flight mass spectrometry; MB-PCR: mutation biased polymerase chain reaction; MCV: mean corpuscular volume; MF: myelofibrosis/myelofibrotic; MWU: Mann-Whitney U; N: number of polycythemia vera patients included in the study; OR: odds ratio; OS: overall survival; PB: peripheral blood; PLT: platelet count; PMN: polymorphonuclear; PRV-1: polycythemia rubra vera 1; qRT-PCR: quantitative real-time polymerase chain reaction; RCM: red cell mass; RR: relative risk; TF: tissue factor; USA: United States of America; VWF: Act: von Willebrand factor activity; VWF: Ag: von Willebrand factor antigen; WBC: white blood cell count; WRS: Wilcoxon rank-sum

Mixed effects models were used for subgroup analyses where applicable. Measures of heterogeneity, including Cochran’s Q, I², and Tau², were reported. Sensitivity meta-analyses were not conducted due to the limited number of publications. In cases where mean and standard deviation were unavailable, the range rule was applied for estimation.

## Results

### Study selection, study characteristics, and risk of bias in studies

**Fig. 1:**
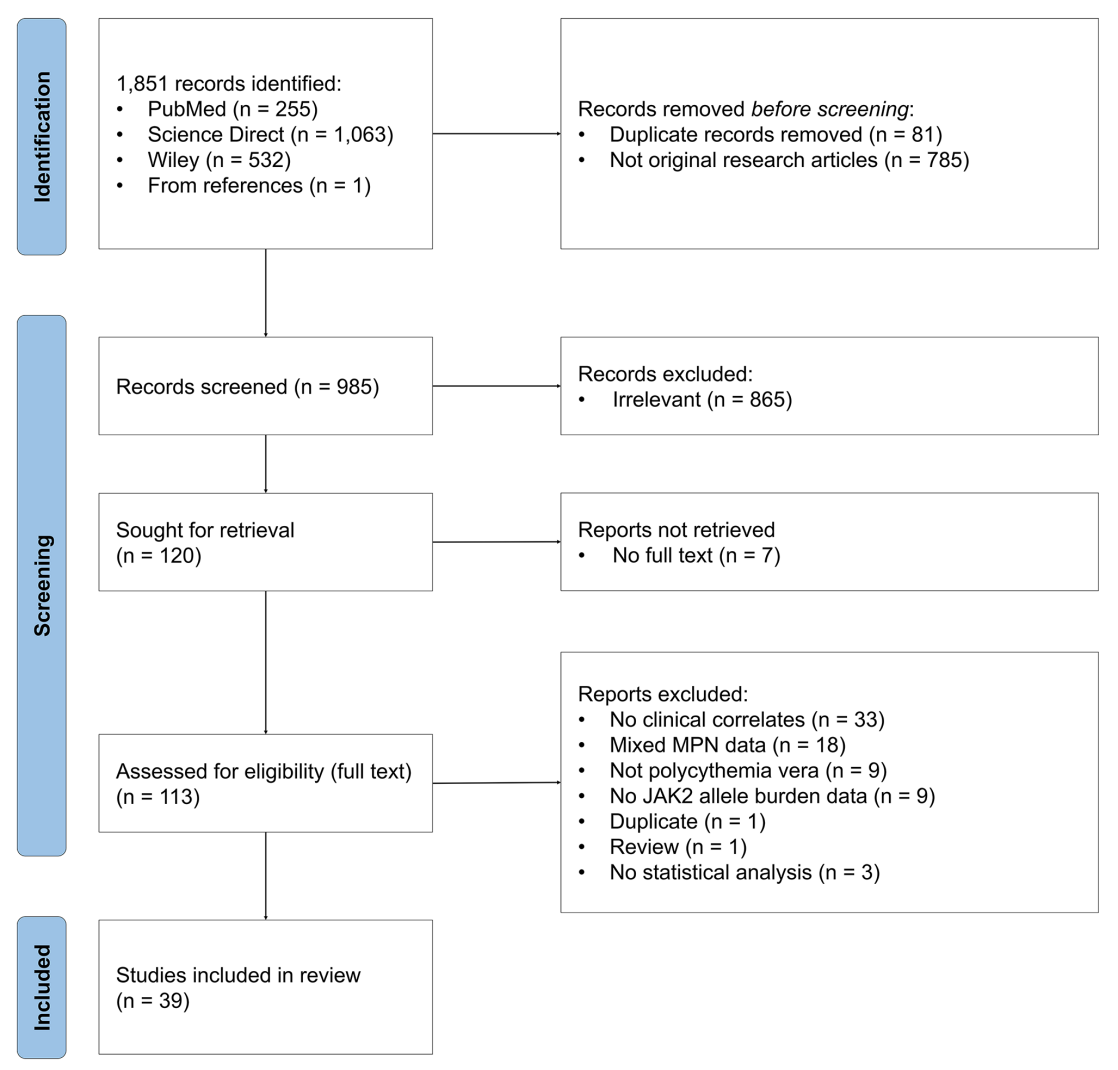
Preferred Reporting Items for Systematic Reviews and Meta-analysis (PRISMA) flow diagram describing the identification, screening, and inclusion process

A flow diagram illustrating the screening process is presented in Fig. 1. Initially, 1,851 studies were identified. After removing duplicates and non-original articles, 985 studies remained. Following title and abstract screening, 120 studies were considered for full-text review. After reviewing the full text, 39 studies [9, 12–49] provided evidence related to the association between *JAK2*V617F allele burden and clinical correlates (Table 1). Details on the excluded 74 records (1 duplicate) [50–122] are presented in Supplemental Information 1.

A total of 21 studies were included in meta-analyses, spanning the years 2006 to 2021 and originating from 12 countries (Belgium, China, Denmark, France, Iran, Italy, Japan, Korea, Macedonia, Spain, Turkey, and the USA). DNA source for *JAK2*V617F allele burden quantification was derived from various cells (e.g., bone marrow, granulocytes, and leukocytes), assessed using different polymerase chain reaction (PCR) and sequencing techniques. Six studies collected samples exclusively at diagnosis, eight studies had a mix of samples at diagnosis and during follow-ups, one study had only follow-up samples, and six studies did not report the collection time point.

Thirteen studies employed correlation tests for *JAK2*V617F with clinical correlates, while ten studies categorized patients into low and high allele burden groups. Notably, despite the initial screening of clinical trials, relevant evidence for our objectives came from cross-sectional and cohort studies, as clinical trials did not investigate the association between clinical characteristics and allele burden. A summary of the risk of bias assessment using the JBI checklist is provided in Supplemental Information 2.

### Meta-analyses of correlation

We examined the correlation of *JAK2*V617F allele burden with blood cell counts and spleen size. WBC and RBC were significantly and positively correlated with *JAK2*V617F allele burden, whereas PLT was not significantly correlated with *JAK2*V617F allele burden. In addition, spleen size was significantly and positively correlated with *JAK2*V617F allele burden.

**Fig. 2:**
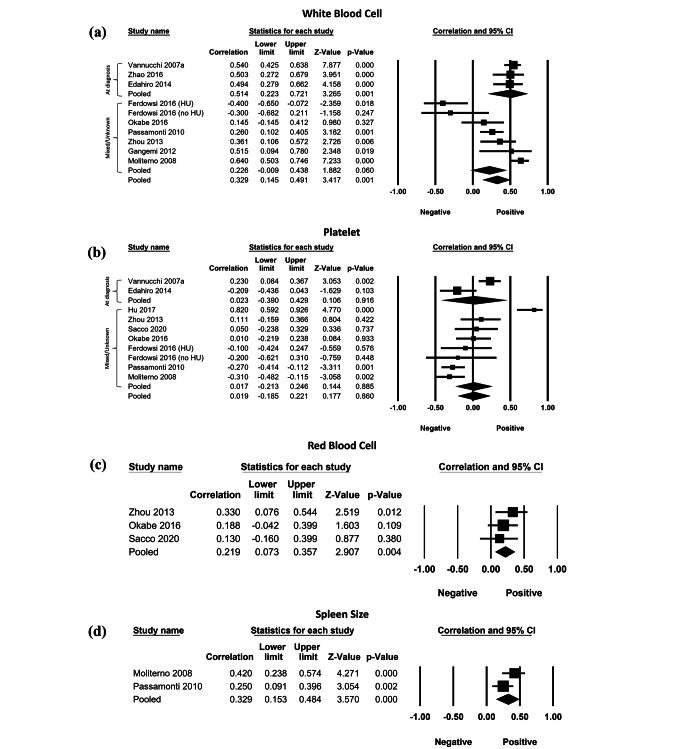
Forest plots of meta-analyses of correlation coefficient of JAK2V617F allele burden vs. (**a**) white blood cell count, (**b**) platelet, (**c**) red blood cell, and (**d**) spleen size. Sub-group analysis was conducted for (a) and (b) to examine the difference between samples collected at diagnosis and those collected at mixed or unstated timepoints

Ten cohorts (703 patients) reported a significant and positive correlation between *JAK2*V617F allele burden and WBC (Fig. 2A: *r* = 0.329; 95%CI=[0.145,0.491]; *p* = 0.001; TauSq = 0.080). Sub-group analysis for the three cohorts (293 patients) with samples collected at diagnosis, demonstrated a significant and positive correlation (*r* = 0.514; 95% CI=[0.223,0.721]; *p* = 0.001). For the seven cohorts (410 patients) with samples collected at mixed or unstated time points, a near-significant and positive correlation was observed (*r* = 0.226; 95% CI=[-0.009,0.438]; *p* = 0.060). Pooled within-group TauSq was 0.077. Total between-group heterogeneity was as follows: Q = 2.526; df = 1; *p* = 0.112.

Ten cohorts (723 patients) demonstrated a non-significant correlation between *JAK2*V617F allele burden and PLT (Fig. 2B: *r* = 0.019; 95%CI=[-0.185,0.221]; *p* = 0.860; TauSq = 0.080). Sub-group analysis for two cohorts (389 patients) with samples collected at diagnosis showed a non-significant correlation (*r* = 0.023; 95% CI=[-0.390, 0.429]; *p* = 0.916). For eight cohorts (334 patients) with samples collected at mixed or unstated time points, a non-significant correlation was observed (*r* = 0.017; 95% CI=[-0.213, 0.246]; *p* = 0.885). Pooled within-group TauSq was 0.088. Total between-group heterogeneity was as follows: Q = 0.001; df = 1; *p* = 0.980.

Three studies (179 patients) reported a significant and positive correlation between *JAK2*V617F allele burden and RBC (Fig. 2C: *r* = 0.219; 95% CI=[0.073,0.357]; *p* = 0.004; TauSq = 0.000). Two studies (240 patients) reported a significant and positive correlation between *JAK2*V617F allele burden and spleen size (Fig. 2D: *r* = 0.329; 95% CI=[0.153,0.484]; *p* < 0.001; TauSq = 0.009). Neither Hb levels nor Hct (Supplemental Information 3, SI3) correlated significantly with allele burden, which respectively included four and two studies.

### Meta-analyses of standardized mean difference

We explored the SMD in clinical correlates between patients with high or low *JAK2*V617F allele burden. WBC, Hct, and lactate dehydrogenase were significantly higher in patients with higher *JAK2*V617F allele burden, whereas PLT was significantly lower in patients with higher *JAK2*V617F allele burden. In addition, Hb and RBC were not significantly different between allele burden groups.

**Fig. 3:**
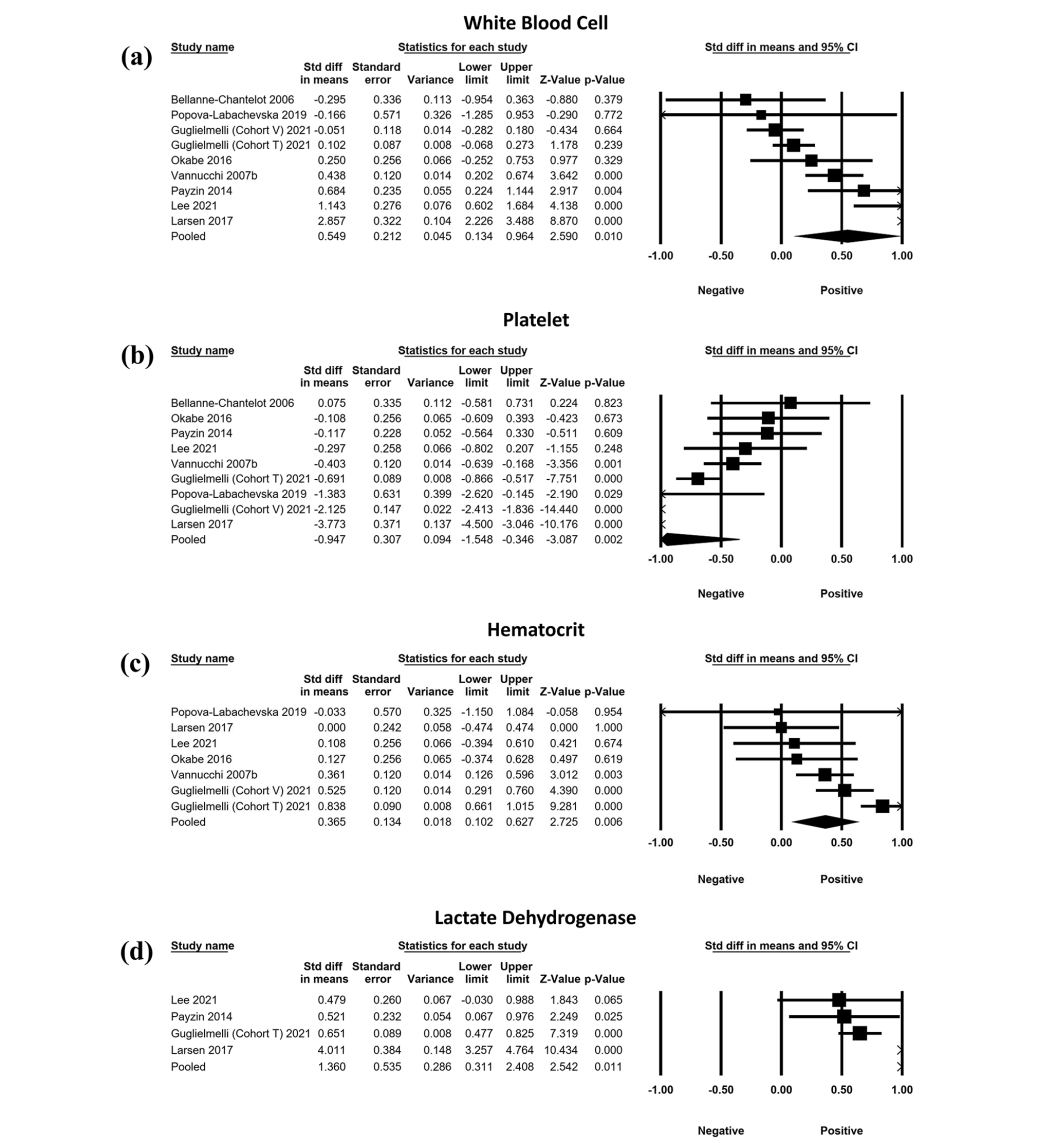
Forest plots of meta-analyses of standardized mean difference of (**a**) white blood cell count, (**b**) platelet, (**c**) hematocrit, and (**d**) lactate dehydrogenase, by allele burden group (high allele burden vs. low allele burden group)

Nine cohorts (1,545 patients) demonstrated a significant SMD, indicating higher WBC in patients with a higher *JAK2*V617F allele burden (Fig. 3A: SMD = 0.549; SE = 0.212; 95%CI=[0.134,0.964]; *p* = 0.010; TauSq = 0.333). Similarly, nine cohorts (1,545 patients) reported a significant SMD in PLT, revealing that patients with a higher *JAK2*V617F allele burden had lower PLT (Fig. 3B: SMD=-0.947.; SE = 0.307; 95%CI=[-1.548,-0.346]; *p* = 0.002; TauSq = 0.762). Seven cohorts(1421 patients) indicated a significant SMD, with higher Hct in patients with a higher *JAK2*V617F allele burden (Fig. 3C: SMD = 0.365; SE = 0.134; 95% CI=[-0.102,0.627]; *p* = 0.006; TauSq = 0.080). Four studies (804 patients) indicated a significant SMD, with higher lactate dehydrogenase in patients with a higher *JAK2*V617F allele burden (Fig. 3D: SMD = 0.1360; SE = 0.535; 95% CI=[0.311,2.408]; *p* = 0.011; TauSq = 1.078). The SMDs of Hb and RBC (SI3) between high and low allele burden groups were not significantly different, involving five and two studies, respectively.

### Meta-analyses of odds ratio

We investigated the odds ratio (OR) of developing symptoms or complications with higher *JAK2*V617F allele burden. We found that patients who had higher *JAK2*V617F allele burden also had a significantly greater OR for developing pruritus, splenomegaly, thrombosis and transformation to MF or AML.

**Fig. 4:**
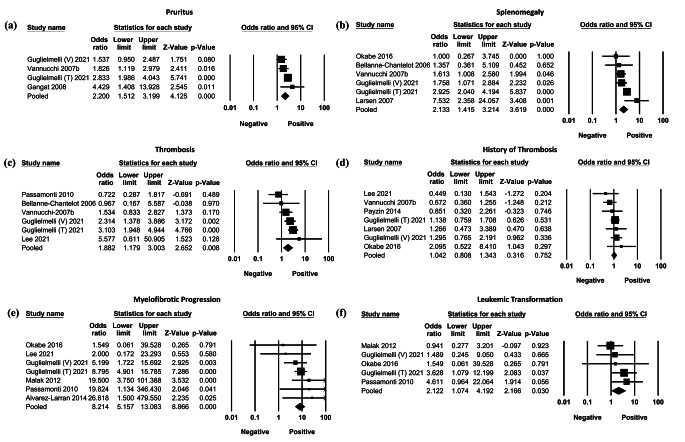
Forest plots of meta-analyses of odds ratio of (**a**) pruritus, (**b**) splenomegaly, (**c**) thrombosis, (**d**) history of thrombosis, (**e**) myelofibrotic progression, (**f**) transformation to acute myeloid leukemia, by allele burden group (high allele burden vs. low allele burden group)

Four cohorts (1,233 patients) reported a significant OR for pruritus, indicating that patients with higher *JAK2*V617F allele burden had a greater OR for developing pruritus (Fig. 4A: OR = 2.200; 95% CI=[1.512,3.199]; *p* < 0.001; TauSq = 0.070). Six cohorts (1,388 patients) reported a significant OR for splenomegaly, indicating that patients with higher *JAK2*V617F allele burden had a greater OR for developing splenomegaly (Fig. 4B: OR = 2.133; 95% CI[1.415,3.214]; *p* < 0.001; TauSq = 0.123). Six cohorts (1,616 patients) reported a significant OR for thrombosis, indicating that patients with higher *JAK2*V617F allele burden had a greater OR for developing thrombosis (Fig. 4C: OR = 1.882; 95% CI=[1.179,3.003]; *p* = 0.008; TauSq = 0.154). *JAK2*V617F allele burden did not seem to significantly affect the odds of thrombosis history in our meta-analyses (Fig. 4D). Seven cohorts (1522 patients) reported a significant OR for MF transformation, revealing that patients with higher *JAK2*V617F allele burden had a greater OR for MF transformation (Fig. 4E: OR = 8.214; 95% CI=[5.157,13.083]; *p* < 0.001; TauSq = 0.000). Five cohorts (1,318 patients) reported a significant OR for AML progression, indicating that patients with higher *JAK2*V617F allele burden had a greater OR for AML transformation (Fig. 4F: 2.122; 95% CI=[1.074,4.192]; *p* = 0.030; TauSq = 0.000).

### Qualitative analysis of patients categorized by clinical characteristics

Several studies examined the *JAK2*V617F allele burden in patients categorized by hemogram thresholds or the presence of a symptom/complication. Consistent with both our meta-analysis on WBC, three studies identified higher *JAK2*V617F allele burdens in PV patients with elevated WBC [40, 41, 47]. As stated above, two studies echoed our finding of a negative association between PLT and *JAK2*V617F allele burden [40, 47].

In terms of splenomegaly, three studies [26, 40, 41] supported our correlation meta-analysis in which spleen size was positively correlated with allele burden. One study [45] found that the risk ratio for splenomegaly and spleen size over 15 cm was higher when comparing patients with low allele burden to patients with high allele burden (0–25% vs. 51–75% and 75–100%); two studies [33, 47] found similar trends between spleen size and allele burden.

For thrombosis, Vannucchi et al. [45] found a significantly higher risk ratio for total thrombosis at follow-up in patients with an allele burden of 75–100% compared to patients with an allele burden of 0–25%. Alvarez-Larran et al. [9] found that patients with *JAK2*V617F allele burden greater or equal to 50% had a higher incidence of thrombosis. Sazawal et al. [40] found that those patients who had experienced a thrombosis event also had a higher allele burden; one study [24] found a similar trend, whereas another study [47] did not.

Finally, Vannucchi et al. [45] found a significantly higher risk ratio for pruritus in patients with 50-75% and 75-100% allele burden. Koren-Michowitz et al. [26] reported a trend for higher allele burden with presence of pruritus; however, this was not significant.

## Discussion

### Interpretation

This review integrates data from 39 publications, encompassing approximately 5,462 patients. To the best of our knowledge, our study represents the first concerted effort to comprehensively evaluate and synthesize the existing literature on the association between *JAK2*V617F allele burden and clinical correlates in PV. Despite over 15 years since the initial discovery of this association, the difficulty of obtaining high-quality data may have impeded previous publications and systematic reviews on this subject. Our combined qualitative analysis and meta-analysis reveal a robust positive association between *JAK2*V617F allele burden and WBC, along with an increased risk of MF transformation. Additionally, positive associations were observed with Hct, RBC, pruritus, splenomegaly, thrombosis, and an increased risk of transformation to AML, while a negative association was noted with PLT.

Our study contributes insights into the association between *JAK2*V617F allele burden and various hematological parameters. The results unequivocally confirm a positive association between *JAK2*V617F allele burden and WBC in PV patients. However, relationships with RBC count, Hct, and Hb levels are less conclusively established. Our meta-analysis of correlation suggests a positive association with RBC, while our meta-analysis of SMD indicates a positive association with Hct, implying some evidence of a positive association between erythrocyte-related parameters and *JAK2*V617F allele burden. Moreover, the intriguing observation of a negative association between PLT and *JAK2*V617F allele burden suggests a potential shift from thrombopoiesis to myelopoiesis when *JAK2*V617F allele burden is elevated, warranting exploration into the biological processes influencing this phenomenon.

Our study explored the association between *JAK2*V617F allele burden and thrombosis, wherein a positive association was observed. Despite the few studies included in meta-analysis, it is crucial to highlight that several independent studies, although could not be synthesized in our meta-analysis, have also presented compelling evidence for a robust association between *JAK2*V617F allele burden and thrombosis [9, 40, 45]. Vannucchi et al. [45] categorized 173 patients into four distinct groups according to their *JAK2*V617F allele burden. They observed that patients with an allele burden of 75% or higher exhibited a significantly elevated risk of thrombosis during the follow-up period. However, due to the scarcity of studies segmenting patients into four groups based on *JAK2*V617F allele burden, a meta-analysis was not feasible. In a similar vein, Alvarez-Larran et al. [9] classified 163 patients into two groups based on their *JAK2*V617F allele burden. Their findings revealed that patients with an allele burden exceeding 50%, or those with fluctuating *JAK2*V617F allele burden, demonstrated a significantly increased incidence of thrombosis. Nevertheless, the absence of comparable studies assessing incidence rates precluded the possibility of conducting a meta-analysis. Additionally, Sazawal et al. [40] stratified 45 patients based on the occurrence of thrombosis events. They found that patients experiencing a thrombosis event had a significantly higher *JAK2*V617F allele burden compared to those without such events. However, the limited number of studies that classified patients based on the occurrence of thrombosis events rendered a meta-analysis unattainable.

Our study also delves into the association between *JAK2*V617F allele burden and symptomatic manifestations as well as disease progression. Concerning spleen size, despite a limited number of studies available for meta-analysis, additional studies [26, 40, 41] reported consistent results, affirming the positive correlation between *JAK2*V617F allele burden and splenomegaly. Similarly, pruritus gains additional validation from another study [26], which reinforces the association between pruritus and *JAK2*V617F allele burden. Furthermore, our study underscores a robust body of evidence linking a high *JAK2*V617F allele burden with an increased risk of MF transformation. This observation posits that elevated *JAK2*V617F allele burden serves as a predictor for MF transformation. Lastly, our study also observed some evidence of positive association between a high *JAK2*V617F allele burden with an increased risk of AML transformation.

In addition to our data synthesis efforts, our investigation reviewed studies that presented valuable insights into the association between *JAK2*V617F allele burden and specific clinical parameters. Notably, a substantial number of studies focused on the relationship between *JAK2*V617F allele burden and splenomegaly, thrombosis, and pruritus, which could have provided further data of 597, 502, and 274 patients, respectively. The majority of these studies consistently reported a statistically significant positive association between *JAK2*V617F allele burden and the aforementioned clinical factors.

### Limitations of evidence and review process

One of the primary constraints in our work stems from the heterogeneity that impeded data synthesis. Despite the identification of 39 studies examining the relationship between the *JAK2*V617F allele burden and clinical correlates, the varied methods of data presentation and statistical analyses prevented the execution of high-quality meta-analyses. For instance, we encountered 16 studies reporting data on allele burden and WBC, of which only 9 could be incorporated into a correlation meta-analysis. Among the remaining 7 papers, data were presented in diverse formats, such as the stratification of data into two to five allele burden groups, and values reported as mean only, mean and range, median and range, median and 95%CI, and mean ± standard deviation. Unfortunately, the inadequate homogeneity across the available studies hindered the synthesis of data, thereby impeding the extraction of conclusive insights.

The reliability of hemogram data may be susceptible to bias owing to the influence of clinical treatments. Among the parameters relevant to erythrocyte count, the most significant variability may arise from phlebotomy. Furthermore, careful consideration is advised when interpreting blood samples obtained during routine check-ups post-diagnosis, as they may be susceptible to underestimation attributed to ongoing treatments such as phlebotomy or the administration of cytoreductive agents. For example, treatment with interferon alpha has been demonstrated to effectively diminish the *JAK2*V617F allele burden, as evidenced by studies from Ianotto et al. [123] and Kiladjian et al. [124]. This reduction in allele burden may subsequently impact the risks associated with thrombosis, myelofibrotic transformation, and leukemic transformation. Consequently, these treatments influence not just the *JAK2*V617F allele burden but also bear significant implications for the long-term outcomes of patients. This complexity adds a layer of challenge to the interpretation of data in this context.

Several assumptions were employed to address heterogeneity during data synthesis. Firstly, heterogeneity arose from the diverse statistical methods used for the meta-analysis of correlation. For example, correlation tests were assumed the same when eleven studies used Spearman’s correlation, four studies used Pearson’s correlation, and two studies did not report the type of correlation test. Secondly, another source of heterogeneity in the meta-analysis of SMD and OR stemmed from the varying cut-off values for *JAK2*V617F allele burden. While the majority of studies divided the patients using a 50% *JAK2*V617F allele burden as a cut-off, one study used 58% [28] and another used 70% [33]. Although a 50% cut-off represents the separation of heterozygosity and homozygosity, using a higher cut-off could better reflect the true impact of *JAK2*V617F allele burden on clinical correlates, such as a more accurate representation of the risk of thrombosis. Thirdly, the inclusion years in our systematic review spanned from 2007 to 2022, during which various diagnostic criteria for PV were utilized, including Polycythemia Vera Study Group (PVSG), World Health Organization (WHO) 2008, and WHO 2016 classification. Consequently, the criteria were not consistent across all studies, and it was assumed that patients diagnosed under different criteria were similar. Lastly, there were differences among studies in the biological samples collected and the methods used to quantify *JAK2*V617F allele burden.

### Implications

Based on our findings, we propose several suggestions for future research aiming to investigate the association between *JAK2*V617F allele burden and clinical correlates. Firstly, detailing the specific time point of sample collection (e.g., at diagnosis, before treatment, or after treatment) is crucial information to include, given the potential impact of certain treatments on *JAK2*V617F allele burden and clinical correlates. Particularly for measurements related to erythrocytes, it is essential to explicitly include Hb, Hct, and RBC without recent phlebotomy, preferably within a three-month timeframe. Attention to the timing of blood sample collection concerning treatment regimens is critical for a more accurate assessment of the relationship between *JAK2*V617F allele burden and hematological parameters across the entire hemogram. Secondly, considering the heterogeneity in study design, data presentation, and statistical methods, the limited amount of data available for synthesis underscores the need for improved feasibility in future meta-analyses. We recommend that researchers consider providing additional data or statistical analyses as supplemental information. Alternatively, utilizing data repositories for sharing relevant datasets could enhance collaboration and facilitate more comprehensive meta-analyses.

This review highlights the varying degrees of association between *JAK2*V617F allele burden and clinical correlates. While some might intuitively infer that reducing *JAK2*V617F allele burden could benefit the status and prognosis of patients, others may argue that a mere observation of association does not necessarily imply a call for action. Nevertheless, there are preliminary data suggesting the potential benefits of reducing *JAK2*V617F allele burden. For instance, a retrospective study involving 381 MPNs patients treated with interferon revealed that approximately 50% of patients who achieved complete hematological response and maintained a *JAK2*V617F allele burden below 10% did not have a relapse for at least ten years after discontinuing interferon treatment [125]. A Phase II clinical trial, MAJIC-PV, comparing ruxolitinib with the best available therapy in patients with PV who are resistant or intolerant to hydroxyurea, demonstrated a higher frequency of molecular responses in those treated with ruxolitinib [126]. Additionally, indirect evidence from molecular analyses and clinical correlations indicates that patients achieving a partial molecular response exhibit improved outcomes in terms of progression-free survival, event-free survival, and overall survival [126]. Another indirect piece of evidence comes from the Continuation-PV study, where patients receiving ropeginterferon alfa-2b demonstrated a general reduction in *JAK2*V617F allele burden and experienced fewer thromboembolic events, less disease progression, and fewer deaths [127]. These findings suggest that novel therapeutic interventions aimed at lowering allele burden could improve not only hemogram but could also manage symptoms, reduce thrombosis risks, and reduce risks of disease progression [128]. Of which, reducing the risks of thrombosis and disease progression are especially important from the perspective of patients [129]. However, a real-world nationwide study in Taiwan showed that around 48.8% low-risk and 26.1% high-risk PV patients were not undergoing active treatment [130]. Additionally, another study in the United States based on a veteran database reported that 53% of patients were not receiving active treatment [131]. As there are some evidence showing that *JAK2*V617F allele burden may progressively increase with age [24, 27, 40, 43, 44], patients without active treatment or monitoring *JAK2*V617F allele burden may be prone to worse outcomes. The rate of clonal expansion exhibits considerable variability among individuals. While some of this variation may be intrinsic, it may also be linked to the type of treatment received by the patient. This relationship underscores the intricate interplay between therapeutic interventions and cellular responses. However, a significant limitation in the current research landscape is the predominance of studies focusing solely on single time point measurements. This methodological constraint restricts the depth of understanding regarding the dynamic nature of clonal expansion over time and its interactions with various treatments. Further research on the clinical value of the long-term monitoring of *JAK2*V617F allele burden could prove valuable in inferring prognosis, guiding monitoring strategies, and designing treatment plans.

This systematic review and its protocol were registered in the international prospective register of systematic reviews (PROSPERO) under the registration number: CRD42024219346.

### Electronic supplementary material

Below is the link to the electronic supplementary material.

Supplementary Material 1

Supplementary Material 2

Supplementary Material 3

## Data Availability

No datasets were generated or analysed during the current study.
