# Supplementary figures and images for: Association of JAK2V617F allele burden and clinical correlates in polycythemia vera: a systematic review and meta-analysis
Source: Ann Hematol. 2024 Apr 23;103(6):1947–65. doi: 10.1007/s00277-024-05754-4 (PMC11090937; doi:10.1007/s00277-024-05754-4)

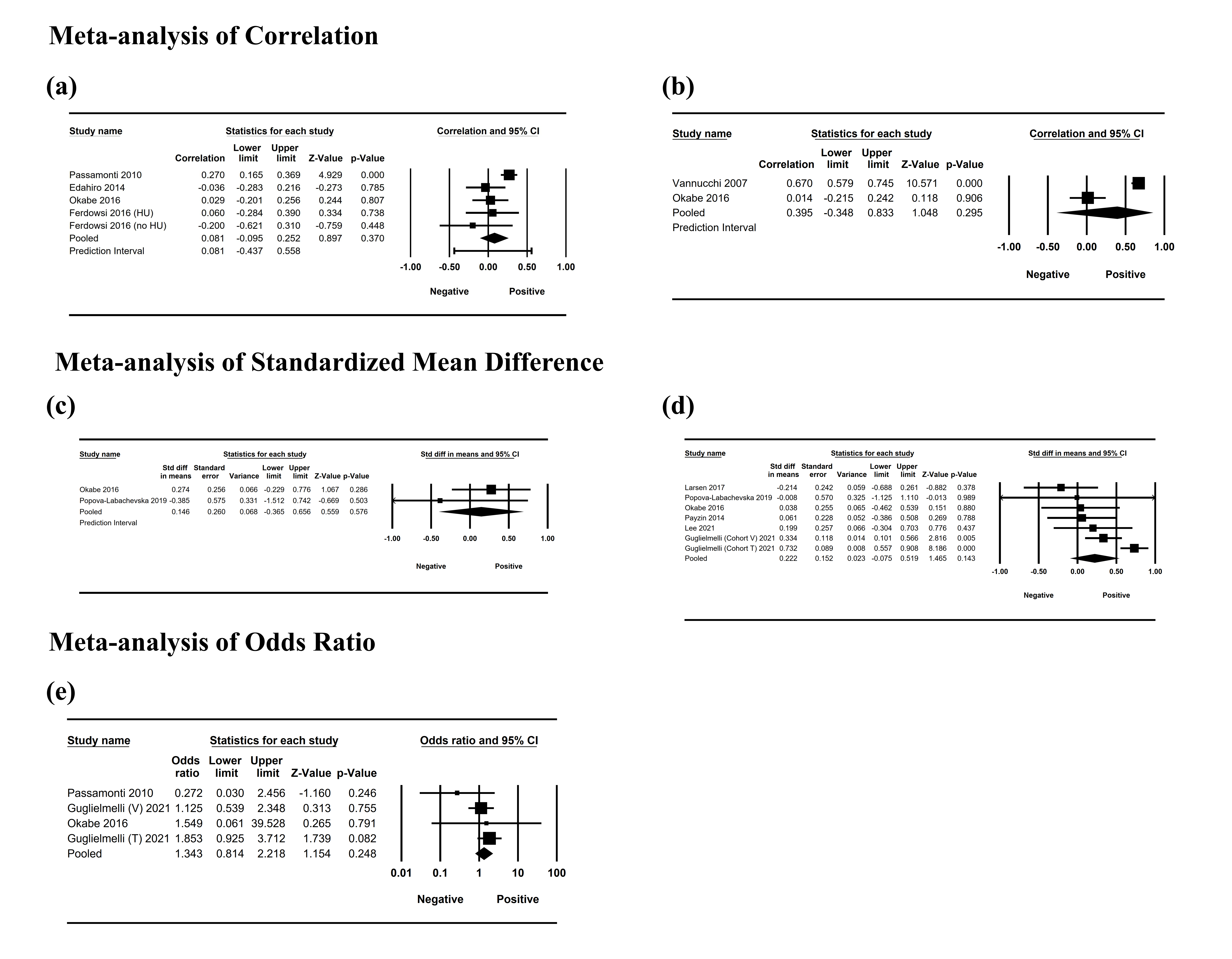

Supplement: Supplementary file 3 — Supplementary Material 3 [file 277_2024_5754_MOESM3_ESM.tif]
